# Durable effects of deep brain ultrasonic neuromodulation on major depression: a case report

**DOI:** 10.1186/s13256-023-04194-4

**Published:** 2023-10-28

**Authors:** Thomas S. Riis, Daniel A. Feldman, Lily C. Vonesh, Jefferson R. Brown, Daniela Solzbacher, Jan Kubanek, Brian J. Mickey

**Affiliations:** 1 Department of Biomedical Engineering, University of Utah, Salt Lake City, USA; 2 Department of Psychiatry, Huntsman Mental Health Institute, University of Utah, Salt Lake City, USA

**Keywords:** Ultrasound, Depression, Neuromodulation

## Abstract

**Background:**

Severe forms of depression have been linked to hyperactivity of the subcallosal cingulate cortex. The ability to stimulate the subcallosal cingulate cortex or associated circuits noninvasively and directly would maximize the number of patients who could receive treatment. To this end, we have developed an ultrasound-based device for effective noninvasive modulation of deep brain circuits. Here we describe an application of this tool to an individual with treatment-resistant depression.

**Case presentation:**

A 30-year-old Caucasian woman with severe treatment-resistant non-psychotic depression was recruited into a clinical study approved by the Institutional Review Board of the University of Utah. The patient had a history of electroconvulsive therapy with full remission but without sustained benefit. Magnetic resonance imaging was used to coregister the ultrasound device to the subject’s brain anatomy and to evaluate neural responses to stimulation. Brief, 30-millisecond pulses of low-intensity ultrasound delivered into the subcallosal cingulate cortex target every 4 seconds caused a robust decrease in functional magnetic resonance imaging blood-oxygen-level-dependent activity within the target. Following repeated stimulation of three anterior cingulate targets, the patient’s depressive symptoms resolved within 24 hours of the stimulation. The patient remained in remission for at least 44 days afterwards.

**Conclusions:**

This case illustrates the potential for ultrasonic neuromodulation to precisely engage deep neural circuits and to trigger a durable therapeutic reset of those circuits.

*Trial registration* ClinicalTrials.gov, NCT05301036. Registered 29 March 2022, https://clinicaltrials.gov/ct2/show/NCT05301036

## Background

Compelling evidence indicates that excessive activity of the subcallosal cingulate (SCC) is associated with depression, and that stimulation of SCC white matter tracts can alleviate symptoms [1–3]. Deep brain stimulation of the SCC white matter has beneficial effects on mood [4], but the surgical risks and the fixed positions of the implanted leads have limited safe and effective applications [5, 6].

Low-intensity transcranial focused ultrasound offers a noninvasive and flexible approach to focally stimulate deep brain structures [7–9]. Ultrasonic neuromodulation holds particular promise for modulation of the deep brain limbic circuits involved in mood disorders.

Proof-of-concept studies of deep brain stimulation with ultrasound to modulate mood have been reported [10–12], but the studies have shown limited effectiveness and effect duration. Two reasons might underlie those results: (1) limited ultrasound intensities delivered into the brain due to the strongly attenuating skull [13, 14] and (2) lack of magnetic resonance imaging (MRI) guidance for precision targeting of specific deep brain targets.

To overcome these limitations, we have developed a new array device, Diadem, that directly measures and compensates for the ultrasound attenuation by the head and hair [14]. This way, the device safely delivers deterministic ultrasound intensity into deep brain targets, which has not been previously possible. We have applied the device, under MRI guidance, to the SCC and associated circuits in a patient with intractable depression. Significantly, a single session of ultrasound stimulation of three SCC-associated targets led to rapid remission of the depressive symptoms. The subject remained in remission at the last assessment 44 days following the treatment.

## Case presentation

The patient is a 30-year-old Caucasian female with severe treatment-resistant depression. The diagnosis was established as a failure to respond to two or more adequate first-line medication treatments. Diagnosis of recurrent major depressive disorder was confirmed with the Mini International Neuropsychiatric Interview (MINI) structured interview (7.0.0). There is a family history of mood disorders, including major depressive disorder, bipolar disorder, and suicide. Onset of depression and anxiety were noted at the age of 13. Between ages 14 and 29 years, she was treated with psychotherapy and underwent medication trials of sertraline, bupropion, citalopram, fluoxetine, duloxetine, trazodone, aripiprazole, quetiapine, clonazepam, lorazepam, lamotrigine, and lithium. She reported initial benefit from most of these agents but less benefit over time; fluoxetine in particular was associated with marked increase of suicidal ideation, which led to her first psychiatric hospitalization. The patient experienced peripartum worsening of depression associated with two live births and one miscarriage. She was hospitalized three times for suicidal ideation. There is no history of attempted suicide, mania, substance use disorder, or psychosis, and no notable medical comorbidities. Her depressive episode reached heightened severity at age 29 [Quick Inventory of Depressive Symptoms self-report (QIDS–SR) score of 16, severe]. She underwent a course of bifrontal electroconvulsive therapy (ECT) and experienced significant improvement with an acute series of eight sessions: QIDS–SR score decreased to 4 (remission) 1 week after the acute series. She had 30 ECT maintenance sessions over the following year. Attempts to reduce the frequency of treatments resulted in recurrence of symptoms. ECT was discontinued due to cognitive and memory problems. At this time the patient was evaluated and enrolled in this study with a six-item Hamilton Depression Rating Scale (HDRS-6) score of 11 and QIDS–SR score of 16. At the time of enrollment and throughout the study, the patient was managed on a combination of bupropion extended release (XL) 450 mg daily, duloxetine 90 mg daily, and lithium extended release (ER) 450 mg twice daily. No changes were made to the patient’s medication regimen during the study. There was no evidence of a developmental or cognitive disorder.

**Fig. 1:**
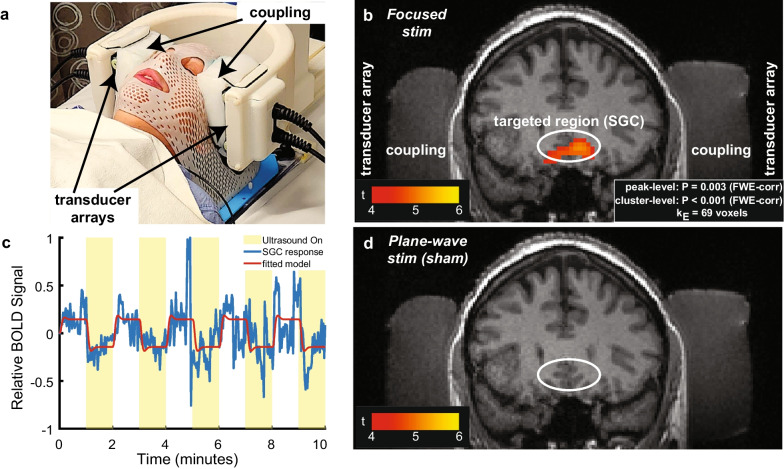
Approach for effective deep brain ultrasonic neuromodulation in humans. **a** Application to a patient with major depression. Programmatic electronic focusing is achieved using two sets of 126 individually controlled transducer elements, one over the left and one over the right side of the head. The subject’s head is secured in place using a standard radiological mask. Lateral windows are made within the mask for unobstructed ultrasound propagation. Coupling is mediated using cryogel. **b** Treatment validation. A standard Siemens flex coil was positioned over the subject’s head. Ultrasound was delivered into the target in 30-millisecond on periods (650 kHz, 1.0 MPa peak pressure) followed by 4-second off periods (0.8% duty). The on and off periods were presented in 1 minute ON blocks, followed by 1 minute OFF blocks of no ultrasound, for a total of up to 10 min (see also **c**). The MRI scanner acquired fMRI BOLD signals during the stimulation. The colorbar shows the t-statistic associated with the BOLD difference between the ON and OFF blocks. The white circles outline the approximate location of the SCC. **c** The modulation of the blood-oxygen-level-dependent (BOLD) signal by the ON and OFF ultrasound conditions. The fitted model (red) assumes standard hemodynamic response. **d** Control stimulation. To control for potential generic artifacts associated with ultrasound, we delivered a stimulus that had the same waveform and pressure amplitude as the stimulus focused into the SCC, but was unfocused (that is, the transducers emitted a plane wave)

**Fig. 2:**
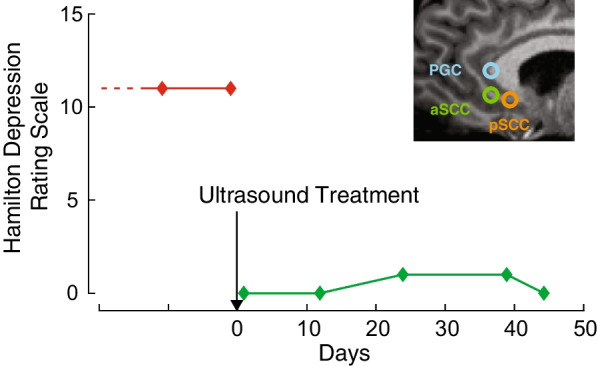
Noninvasive deep brain stimulation is capable of improving mood states in patients with major depression. Following a single treatment session of 64-minutes of active stimulation to three separate targets of the SCC, the subject’s HDRS-6 score fell from 11 to 0. Depression remained in remission for the 44 days while the subject was monitored, with a final HDRS-6 of 0. Inset shows the SCC targets sonicated

The objective of this case report is to describe application of low-intensity transcranial focused ultrasound to an individual with treatment-resistant depression. We validated the engagement of the modulated target, SCC, with functional MRI (Fig. 1) and report the progression of HDRS-6 scores up to 44 days following the stimulation (Fig. 2).

The ultrasonic stimulation array, Diadem, consists of two sets of ultrasound transducer arrays positioned at opposite sides of the head [15]. This configuration enables Diadem to electronically focus the ultrasound into specified deep brain targets and compensate for the ultrasound attenuation by the skull, hair, and coupling media [14].

To evaluate the immediate effects of the stimulation on mood states, we used Diadem to modulate three separate areas of the cingulate cortex in this subject over a 2-hour stimulation session. The intensity field delivered into the brain had lateral × elevational × axial dimensions of 2.4 mm × 3.6 mm × 20.4 mm [*y*, *z*, and *x* dimensions of the Montreal Neurological Institute (MNI) coordinate system [15]]. The targets were centered on posterior SCC [16] [MNI coordinate (0, 26.21, −8.11) (*x*, *y*, *z* from MNI center coordinate)], anterior SCC [MNI coordinate (0, 34.21, −6.11)], and pregenual cingulate [MNI coordinate (0, 34.21, 3.11)]. These targets were chosen to maximize the probability of modulating white matter tracts within SGC [4]. Each target was sonicated with a 650 kHz continuous wave for 30-millisecond ON periods followed by 4-second OFF periods (0.8% duty) for an average duration of 2 minutes (range 20–180 seconds). The estimated peak pressure at target was 1.0 MPa following Diadem’s compensation for the ultrasound attenuation by the head and hair [14]. Each target was sonicated ten times, with randomized order between the three sites, for a total of 30 stimulation epochs spanning 64 minutes of active stimulation. The stimulation intensity was maintained below the US Food and Drug Administration (FDA) 510(k) Track 3 guidelines (peak intensity < 190 W/cm^2^ and time-averaged intensity < 720 mW/cm^2^).

To evaluate any durable effects of the stimulation, we collected HDRS-6 scores before and after the stimulation [17]. The presonication HDRS-6 score of 11 fell to 0 the day following the stimulation, indicating effective remission (Fig. 2). On the day following treatment, the subject reported, “This is the first time in three years I have felt like myself; it feels like my brain has been woken up.” The effects were durable and the patient remained in remission (HDRS-6 = 0) for at least 44 days following the sonication, the last assessed timepoint. About 5 months after the stimulation, she started to notice a recurrence of the depression; medications were continued unchanged during the 5 month interval.

We evaluated the safety of the stimulation at the behavioral and anatomical levels. At the behavioral level, the subject completed a standard clinical questionnaire of stimulation side effects [18]. No adverse effects were noted by the subject or the attending psychiatrist. Subject completed the General Assessment of Side Effects (GASE) survey and reported no side effects related to treatment (Table 1). Moreover, no anomalies were observed on either T1-weighted or T2-weighted MRIs of the subject’s brain.

**Table 1:** The stimulation was safe without adverse effects

Adverse effects related to treatment	Session 1	Session 2	Session 3	Adverse effects related to treatment	Session 1	Session 2	Session 3
Headache	No	No	No	Skin rash or itching	No	No	No
Dry mouth	No	No	No	Tendency to develop bruises	No	No	No
Dizziness	No	No	No	Fever, increased temperature	No	No	No
Chest pain	No	No	No	Abnormal sweating	No	No	No
Palpitations	No	No	No	Hot flashes	No	No	No
Breathing problems	No	No	No	Convulsions or seizures	No	No	No
Circulation problems	No	No	No	Fatigue, loss of energy	No	No	No
Abdominal pain	No	No	No	Tremor	No	No	No
Nausea	No	No	No	Insomnia, sleeping problems	No	No	No
Vomiting	No	No	No	Back pain	No	No	No
Constipation	No	No	No	Muscle pain	No	No	No
Diarrhea	No	No	No	Joint pain	No	No	No
Reduced appetite	No	No	No	Agitation	No	No	No
Increased appetite	No	No	No	Irritability, nervousness	No	No	No
Difficulty urinating	No	No	No	Depressed mood	No	No	No
Sexual problems	No	No	No	Thoughts about suicide	No	No	No
Painful or irregular menstruation	No	No	No	Anxiety, fearfulness	No	No	No

Following the stimulation, the patient was asked to complete a clinical questionnaire that assessed potential side effects. The options were “No,” “Maybe,” and “Yes”

## Discussion and conclusions

We report rapid and sustained improvement in depression following direct ultrasonic modulation of deep brain targets associated with the SCC. The stimulation was followed by remission lasting for at least 6 weeks. No safety concerns or side effects were noted.

The approach provides three notable strengths over existing neuromodulation devices in that it (1) delivers stimulation noninvasively into deep brain targets, (2) provides precise and flexible electronic targeting, and (3) delivers controlled stimulation intensity into the targets [14].

Using functional MRI (fMRI), we further demonstrated that the device significantly and substantially engaged the specified deep brain target, the SCC, and its associated circuits. The stimulation resulted in a significant decrease in fMRI BOLD activity at the target, which suggests an inhibition of the SCC. This effect was only observed during active stimulation and not during sham stimulation.

This finding illustrates the potential of transcranial focused ultrasound as a powerful modality for direct and durable reset of malfunctioning circuits. However, this case report cannot demonstrate causality. This approach must be validated in future randomized controlled clinical trials that include a corresponding sham. Appropriate sham stimuli are beginning to be developed for transcranial focused ultrasound [19].

The approach is not limited to modulation of the SCC; the ultrasonic array device presented here is capable of modulating targets throughout the deep brain [15]. For instance, the device could target the ventral posteromedial or ventral posterolateral nuclei of the thalamus in patients with chronic pain [20, 21].

Transcranial low-intensity ultrasound has been safely applied to human subjects in previous studies [12, 22, 23], but the strongly aberrating properties of the skull have severely limited the predictability of the delivered intensity [24]. Uncertainty associated with transcranial ultrasound delivery could raise safety concerns, since an overcompensation for the ultrasound attenuation of the skull could lead to mechanical or thermal tissue damage. Diadem measures the acoustic properties of an individual’s skull and hair using a through-transmit scan and adjusts the delivered ultrasound stimuli accordingly [14], enabling the operator to effectively deliver stimulation that remains within well-established safety limits. The ultrasound intensity delivered in this study is limited to the FDA 510(k) Track 3 safety guidelines for safe ultrasound imaging [25]: the spatial peak temporal average intensity of less than 0.72 W/cm^2^, and the spatial peak pulse average intensity less than 190 W/cm^2^.

It is now established that ultrasound of sufficient duration and intensity induces durable neuroplastic effects in the target circuits [11, 12, 26–32]. These effects are believed to be mediated, at least in part, by activation of glial cells and the ensuing effects on synaptic processes [33]. This and related molecular pathways provide unique opportunities for durable circuit reset, akin to electroconvulsive therapy or repeated applications of transcranial magnetic stimulation, but now applied in a targeted manner and directly to the involved deep brain circuits. This approach is expected to increase the effectiveness and safety of neuromodulation treatments, providing targeted patient-specific reset of the malfunctioning deep brain circuits.

## Data Availability

Deidentified patient data will be provided upon reasonable request from the corresponding author.
